# Correlation between echocardiographic estimation of right atrial pressure and invasive measurement of central venous pressure in postoperative pediatric patients with congenital heart disease: a prospective observational study

**DOI:** 10.1186/s43044-024-00456-8

**Published:** 2024-02-21

**Authors:** Elaheh Malakan Rad, Nazli Parizadeh, Hassan Radmehr, Toktam Sheykhian, Behdad Gharib, Aliakbar Zeinaloo

**Affiliations:** 1 Children’s Medical Center (Pediatric Center of Excellence), Affiliated with Tehran University of Medical Sciences, No. 62, Dr. Gharib’s Street, End of Keshavarz Boulevard, Tehran, 1419733151 Iran; 2 Imam Khoemoeini’s Hospital, Affiliated with Tehran University of Medical Sciences, No. 62, Dr. Gharib’s Street, End of Keshavarz Boulevard, Next to Children’s Medical Center, Tehran, 1419733134 Iran

## Abstract

**Background:**

Right atrial pressure plays a critical role as a hemodynamic parameter in diagnosing pulmonary hypertension and other cardiac diseases, as well as guiding the treatment and prognosis of various cardiac disorders. If there is no obstruction between the inferior or superior vena cava (SVC) as central veins and the right atrium, the pressures in these veins could be considered equal to the right atrial pressure. This study aimed to examine the correlation between echocardiographic methods for estimating right atrial pressure and invasive measurements of central venous pressure (CVP_i_) in infants and children with congenital heart disease during the 48 h after cardiac surgery and to establish regression equations for echocardiographic estimation of central venous pressure (CVP_e_).

**Results:**

We prospectively enrolled 43 infants and children, ranging in age from 6 months to 16 years, including 20 males and 23 females. We found a significant correlation between CVP_i_ and the ratio of the maximal diameter of IVC to the maximal diameter of the descending aorta ratio (IVC_max_/DAO_max_) (*r* = 0.529, *P* < 0.001), SVC_S/D_ velocity ratio (SVC_S/D_) (*r* = 0.462, *P* = 0.006), right atrial vertical diameter (RA_VD_) (*r* = 0.409, *P* = 0.01), area (*r* = 0.384, *P* = 0.014), and tricuspid valve A wave acceleration rate (TV_AAR_) (*r* = 0.315, *P* = 0.048). Multiple regression analysis yielded an equation for estimating central venous pressure using four parameters related to the IVC, SVC, tricuspid valve, and right atrium. The equation is as follows: estimated CVP = 4.36 + (2.35 × IVC_max_/DAO_max_) + (1.06 × SVC_S/D_) +  (0.059 × RA_VD_) + (0.001 × TV_AAR_). This equation is strongly correlated with CVP_i_ (Pearson *r* = 0.698, *P* = 0.002).

**Conclusions:**

The estimation of central venous pressure through a multi-parametric equation that included the ratio of the maximal diameter of the inferior vena cava to the maximal diameter of the descending aorta, the ratio of *S* to *D* velocity of the superior vena cava, the vertical diameter of the right atrium, and the acceleration rate of the A wave of the tricuspid valve demonstrated a robust correlation with invasively measured central venous pressure. To assess the accuracy of predicted pressures by this equation, further investigations are required to apply this innovative multi-parametric formula to a prospective population of pediatric patients with congenital heart disease.

## Background

Right atrial pressure plays a critical role as a hemodynamic parameter in diagnosing pulmonary hypertension and other cardiac diseases, as well as guiding the treatment and prognosis of various cardiac disorders [1–5]. In the absence of any obstruction between the inferior or superior vena cava and the right atrium, it is reasonable to assume that the pressures in these central veins are equal to that of the right atrium [6]. Limited data exist regarding the performance of different echocardiographic methods for estimating right atrial pressure in infants and children with congenital heart disease, especially within the first 48 h following cardiac surgery in the intensive care unit setting. The objectives of this study were to assess the correlation between echocardiographic methods for estimating right atrial pressure and invasive measurements of central venous pressure in infants and children with congenital heart disease during the 48-h postoperative period and to establish regression equations for the echocardiographic estimation of central venous pressure in this particular context.

## Methods

### Study design and study population

We conducted a prospective observational study between 2021 and 2022, enrolling infants and children who had undergone cardiac surgery for congenital heart disease within the first 48 h after the operation, provided that specific conditions were met. These conditions included invasive monitoring of central venous and arterial pressures through catheterization of the internal jugular vein and femoral artery, absence of obstruction between the inferior vena cava (IVC) and the right atrium, exclusion of Glenn bidirectional shunt or Fontan operation as surgical procedures, extubated with spontaneous breathing, presence of an adequate acoustic window for echocardiographic examination, and maintenance of stable hemodynamic status in the patient. During echocardiography, all patients were awake and in a calm state.

### Measurement of central venous pressure

The central venous pressure was measured using Arrow pediatric three-lumen central venous catheter (Arrow International LLC, Morrisville, NC 27560 USA) inserted into the internal jugular vein and Bioptimal disposable pressure monitoring kit (Biosensors International, Shanghai International Holding Corp. 20,537 Hamburg, Germany). The positioning accuracy of the central venous catheter was assessed by examining the chest X-ray of every patient [4].

The measurements were standardized.

### Echocardiographic examination

The echocardiographic examinations were performed in the intensive care unit using the Philips Affinity 70 C echocardiography machine (Philips Healthcare, USA), equipped with probes operating at 5 and 8 megahertz (MHz) frequencies.

All echocardiographic examinations were conducted by a senior and well-trained fellow of pediatric cardiology, with the patient positioned in a supine posture. Standard echocardiographic images were acquired from various windows, including subcostal, apical, parasternal, and suprasternal views.

**Fig. 1:**
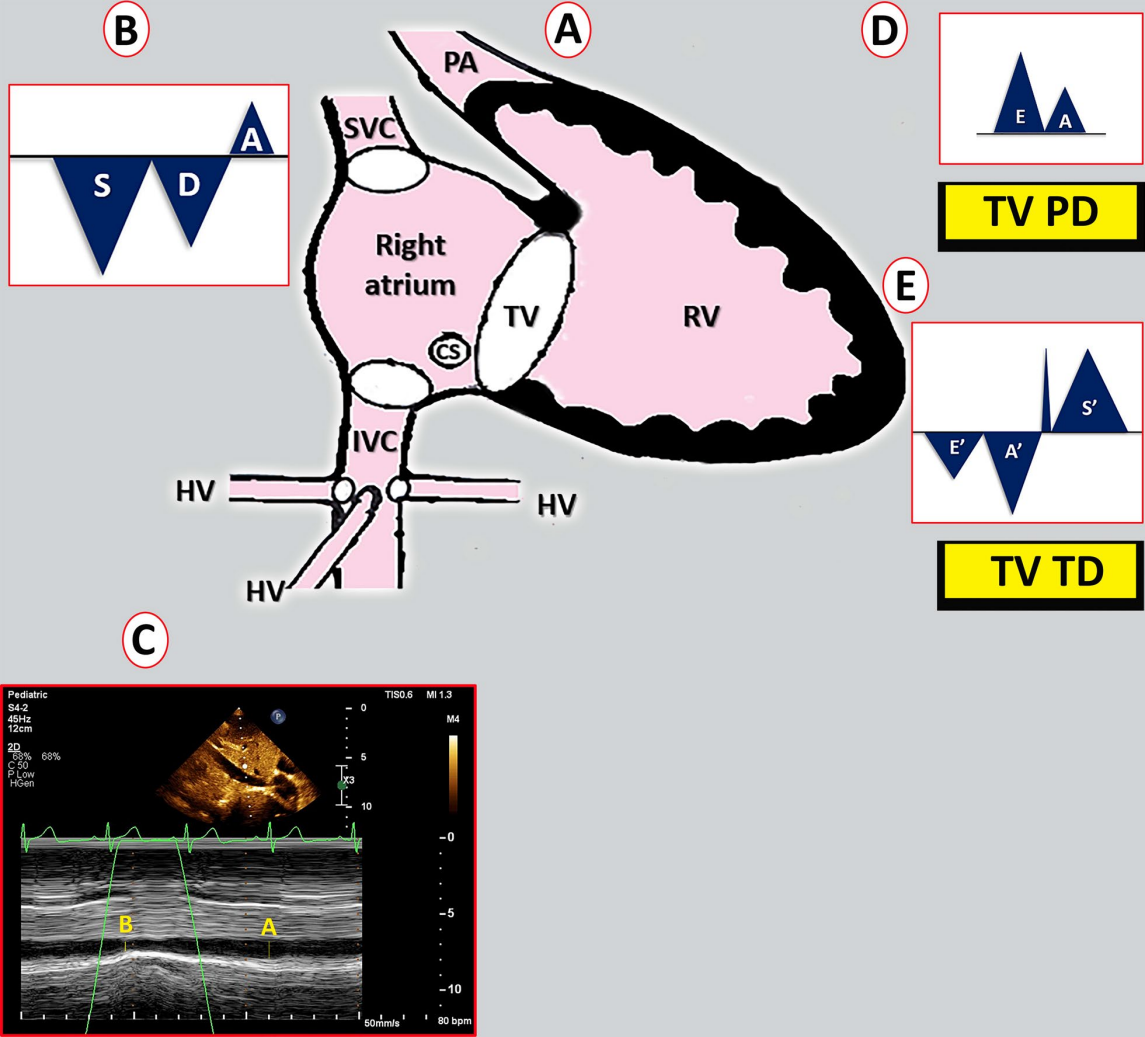
**A** The heart and the connections of the right atrium to the surrounding vessels. **B** The Doppler profile of the inferior vena cava, superior vena cava, and hepatic vein. **C** The measurement of maximal and minimal diameters of the inferior vena cava. **D** The pulse-wave Doppler of the tricuspid valve flow. **E** The tissue Doppler of the lateral annulus of the tricuspid valve (*IVC* inferior vena cava, *SVC* superior vena cava, *HV* hepatic vein, *TV* tricuspid valve, *PW* pulse Doppler, *TD* tissue Doppler)

In the right ventricular focused four-chamber view, measurements of Doppler variables related to the tricuspid valve and dimensions of the right atrium were obtained in accordance with established guidelines [7–9]. Doppler evaluation of the hepatic vein and inferior vena cava, as well as the measurement of the maximum and minimum diameter of the inferior vena cava at a proximal point to the junction of the hepatic vein, and descending aorta at the same level, was conducted in the subcostal window, following the previously described methodology. The superior vena cava was examined from the suprasternal window [10–12] (Fig. 1). The measurements were standardized.

The diameters of the inferior vena cava and right atrial volume were indexed by the body surface area. The ratio between the minimum and maximum diameters of the inferior vena cava (IVC) to the minimum and maximum diameters of the descending aorta (DAO) at the same level and inferior vena cava collapsibility and distensibility indices is calculated using the following formulas [13]:

$$\frac{{{\text{IVC}}_{{{\text{max}}}} }}{{{\text{DAO}}_{{{\text{max}}}} }} = \frac{{\text{Maximal diameter of inferior vena cava}}}{{\text{Maximal diameter of descending aorta}}}$$

$$\frac{{{\text{IVC}}_{{{\text{min}}}} }}{{{\text{DAO}}_{{{\text{min}}}} }} = \frac{{\text{Minimal diameter of inferior vena cava}}}{{\text{Minimal diameter of descending aorta}}}$$

$${\text{IVC collapsibility index}} = \frac{{{\text{IVC maximal size in inspiration}} - {\text{IVC minimal size in expiration }}}}{{\text{IVC maximal size in inspiration}}}$$

$${\text{IVC distensibility index}} = \frac{{{\text{IVC maximal size in inspiration}} - {\text{IVC minimal size in expiration }}}}{{\text{IVC minimal size in inspiration}}}$$

### Statistical analysis

The data distribution was evaluated using the Shapiro–Wilk test to assess its normality. Descriptive statistics, including the mean, standard deviation, median, interquartile range, minimum, and maximum, were provided for continuous variables. The presentation of categorical variables included the absolute counts and corresponding percentages.

The correlation between invasively measured central venous pressure and numerical echocardiographic variables was evaluated using linear regression, and Pearson r correlation coefficient and R square values were reported. The Chi-square or Fisher’s exact test assessed the association between categorical variables. The statistical analysis was conducted using IBM SPSS Statistics for Windows, version 27 (IBM Corp., Armonk, NY, USA). A significance level of *P* < 0.05 was used to determine statistical significance.

### Ethical considerations

Informed parents or guardians’ consent was obtained. This study received approval from the Institutional Research Ethics Committees. The study was conducted in accordance with the ethical guidelines outlined in the 2013 Declaration of Helsinki [14].

## Results

**Fig. 2:**
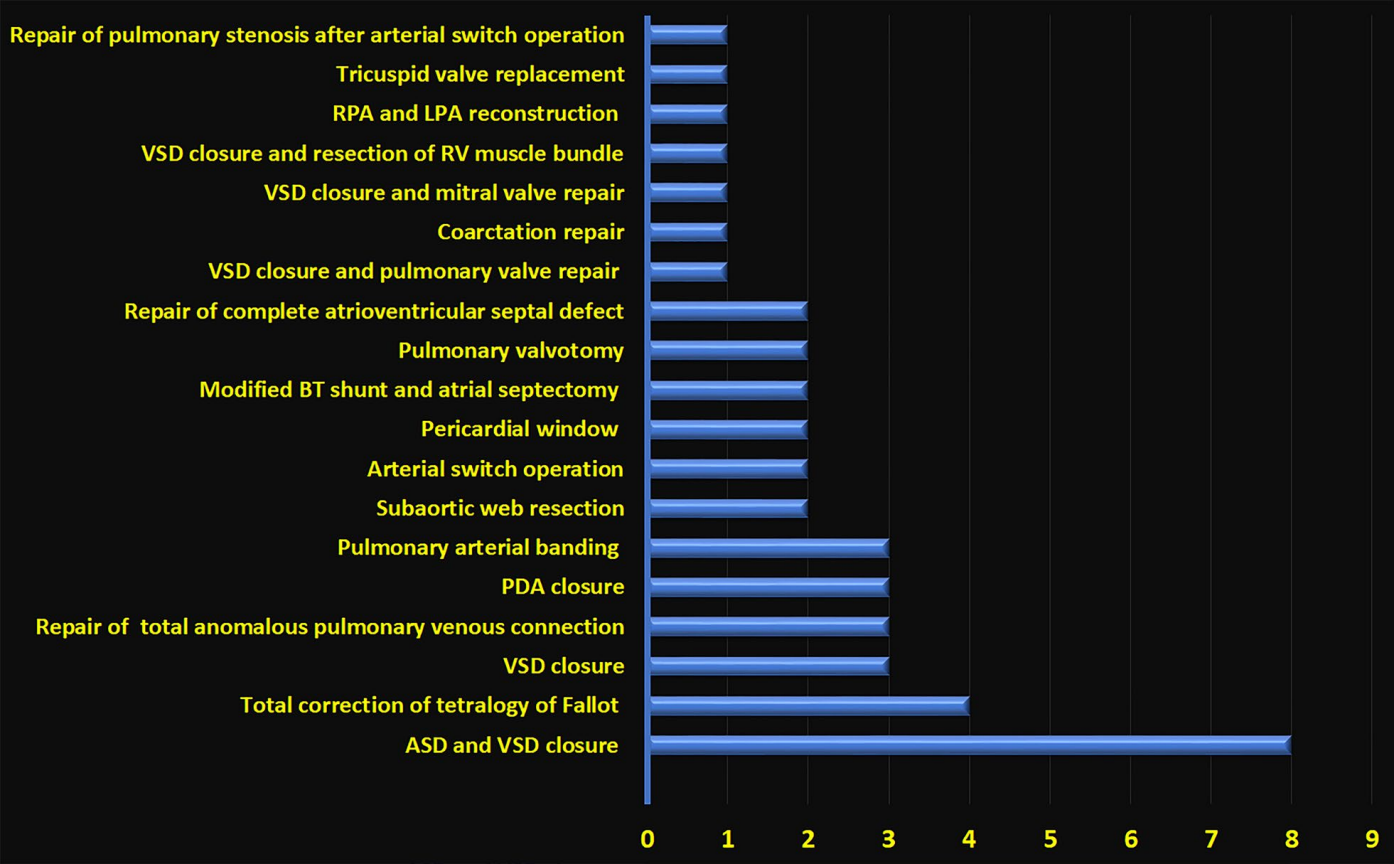
This figure shows the diagnoses of 43 patients. *ASD* atrial septal defect, *BT* Blalock–Taussig, *LPA* left pulmonary artery, *PDA* patent ductus arteriosus, *RPA* right pulmonary artery, *RV* right ventricle, *VSD* ventricular septal defect

We prospectively enrolled 43 infants and children, ranging in age from 6 months to 16 years. The study included 20 males and 23 females. The diagnoses of the patients are shown in Fig. 2. Table 1 represents a descriptive analysis of the basic characteristics and variables. Tables 2 and 3 provide a descriptive analysis of the measured and calculated variables for the following structures: inferior and superior vena cava, hepatic vein, tricuspid valve, and right atrium.

**Table 1:** Basic characteristics of the study population

Total number of the study population	43
Male (number, frequency)	20, 46.5%
Female (number, frequency)	23, 53.5%

Basic variables	Mean	Standard deviation	Minimum	Maximum	Median	Interquartile range
*(IQR)*						
Age (months)	28.61	43.09	0.50	192.00	9.00	45
Weight (kilograms)	10.28	9.45	2.40	41.00	6.30	7.25
Height (centimeter)	77.65	26.55	47.00	140.00	65.00	38.50
Body surface area (square meters, m^2^)	0.46	0.28	0.18	1.26	0.34	0.27
Heart rate (beats per minute)	128.50	21.32	71.00	165.00	127.50	34.00
Blood pressure, systolic (mmHg)	94.91	17.60	60.00	140.00	67.00	23.00
Blood pressure, diastolic (mmHg)	55.40	12.88	31.00	93.00	96.00	15.00
Blood pressure, mean (mmHg)	69.05	13.88	44.00	111.00	53.00	18.00
Central venous pressure (mmHg)	10.81	2.64	8.00	17.00	10.00	3.50

**Table 2:** Descriptive analysis of the measured variables of the study (in alphabetical order)

Variables	Unit	Minimum	Maximum	Mean	Standard deviation	Median	Interquartile range (IQR)
Days, postoperative	Days	1	6	3.44	1.42	4	2.25
Descending aorta, maximal diameter	Millimeters	4	13	6.87	1.81	6.55	2
Descending aorta, minimal diameter	Millimeters	2.5	10	4.9	1.62	4.3	2.15
*1. Hepatic vein parameters*							
Hepatic vein antegrade diastolic velocity (D wave)	Centimeters/seconds	0	70.1	37.59	14.98	35.65	26.35
Hepatic vein antegrade diastolic velocity time integral (D wave)	Centimeters	0	16	5.65	3.74	5.28	4.21
Hepatic vein antegrade systolic velocity (S wave)	Centimeters/seconds	17.6	121	47.06	23.20	40.95	27.6
Hepatic vein antegrade systolic velocity time integral (S wave)	Centimeters	1.57	33	8.00	6.33	6.35	6.5
Hepatic vein maximal forward velocity	Centimeters/seconds	1.9	13	5.75	2.33	5.45	2.78
Hepatic vein minimal forward velocity	Centimeters/seconds	1	10	4.05	2.08	3.75	2.63
Hepatic vein reversal flow velocity (A wave)	Centimeters/seconds	12.4	72	32.05	13.07	30	18.5
Hepatic vein reversal flow velocity time integral (A wave)	Centimeters	1.16	7.83	3.42	1.80	2.77	2.95
Hepatic vein V Wave velocity	Centimeters/seconds	0	34.7	10.76	11.81	9.6	20.05
Hepatic vein V Wave velocity time integral	Centimeters	0	5.5	0.93	1.38	0.5	1.54
*2. Inferior vena cava parameters*							
Inferior vena cava systolic flow velocity	Centimeters/seconds	15.3	100	51.21	20.12	46.18	27.05
Inferior vena cava systolic flow velocity time integral	Centimeters	2.58	29.2	8.49	5.26	6.98	4.82
Inferior vena cava diastolic velocity	Centimeters/seconds	18.3	120	40.18	19.64	35	24.85
Inferior vena cava diastolic velocity time integral	Centimeters	1.45	45	7.17	7.43	5.47	3.90
Inferior vena cava reversal flow velocity	Centimeters/seconds	0	43.6	23.90	8.93	21.7	13.15
Inferior vena cava reversal flow velocity time integral	Centimeters	0	7.8	2.65	1.83	2.25	1.94
Inferior vena cava, maximal diameter	Millimeters	2	18	6.67	3.35	6.1	3.4
Inferior vena cava, minimal diameter	Millimeters	1.3	10.2	4.33	2.12	3.8	2.95
*3. Right atrial parameters*							
Right atrial horizontal diameter	Millimeters	11.7	36.7	21.33	5.97	22.55	8.93
Right atrial vertical diameter	Millimeters	14	45.5	24.39	7.01	24.6	8.70
Right atrial area	Centimeters^2^	1.71	12.8	5.04	2.44	5.48	3.92
Right atrial volume	Centimeters^3^	1.53	31.9	9.60	6.70	9.12	9.22
Body-surface area-indexed right atrial volume	Centimeters^3^/m^2^	6.34	49.02	18.25	9.78	15.78	10.98
*4. Superior vena cava parameters*							
Superior vena cava systolic velocity	Centimeters/seconds	49.5	130	78.43	19.82	76.15	26.17
Superior vena cava systolic velocity time integral	Centimeters	4.93	31.30	13.30	6.89	11.1	5.67
Superior vena cava diastolic velocity	Centimeters/seconds	22.4	96.50	56.75	20.14	55.09	28.80
Superior vena cava diastolic velocity time integral	Centimeters	3.18	25.11	8.66	5.55	7.3	5.73
Superior vena cava reversal velocity	Centimeters/seconds	8.5	62.5	26.17	12.06	23.95	14.73
Superior vena cava reversal velocity time integral	Centimeters	0.48	7.87	2.56	1.71	2.03	2.41
*5. Tricuspid valve parameters*							
Tricuspid valve A velocity	Centimeters/seconds	26.1	86.9	52.42	19.71	49	41.85
Tricuspid valve A velocity time integral	Centimeters	1.59	9.8	4.61	2.25	4.29	2.47
Tricuspid valve A' wave	Centimeters/seconds	2.32	9.40	5.34	1.93	5.1	2.68
Tricuspid valve A wave, acceleration rate	Centimeters/second^2^	375	7958	1419.42	1203.67	1206	839
Tricuspid valve A wave, acceleration time	Milliseconds	8	92	43.61	19.62	42	27.35
Tricuspid valve A wave, deceleration rate	Centimeters/second^2^	143	1966	785.46	446.78	682	672
Tricuspid valve A wave, deceleration time	Milliseconds	21	187	71.75	31.66	63	4305
Tricuspid valve A wave, duration	Milliseconds	45	201	111.26	38.68	106	62.5
Tricuspid valve E velocity	Centimeters/seconds	42.2	143	77.91	24.72	72.9	34.55
Tricuspid valve E velocity time integral	Centimeters	3.37	16.9	7.44	2.98	6.9	4.09
Tricuspid valve E′ wave	Centimeters/seconds	2.92	28.7	9.62	5.06	8.3	4
Tricuspid valve E wave, acceleration rate	Centimeters/second^2^	727	3772	1640.15	730.43	1506	882
Tricuspid valve E wave, acceleration time	Milliseconds	11	111	52.17	18.99	50	23.5
Tricuspid valve E wave, deceleration rate	Centimeters/second^2^	191.18	2272	1007.90	251.87	934	520.75
Tricuspid valve E wave, deceleration time	Milliseconds	33	244	85.48	38.25	74	3
Tricuspid valve *E* wave, duration	Milliseconds	69	193	122.86	31.16	111	51
Tricuspid valve isovolumic relaxation time	Milliseconds	0	87	23.58	27.82	30	49

**Table 3:** Descriptive analysis of the calculated variables of the study

Variables	Unit	Minimum	Maximum	Mean	Standard deviation	Median	Interquartile range (IQR)
*Hepatic vein*							
Hepatic vein systolic velocity/diastolic velocity	–	0.47	2.25	1.30	0.53	1.37	0.98
Hepatic vein systolic filling fraction	–	0.24	1	0.58	0.17	0.57	0.26
*Inferior vena cava*							
Inferior vena cava collapsibility index	–	0.08	0.64	0.34	0.16	0.34	0.24
Inferior vena cava distensibility index	–	0.09	1.80	0.64	0.47	0.51	0.57
*Superior vena cava*							
S wave velocity/*D* wave velocity ratio	–						
Inferior vena cava *S*/*D* ratio	–	0.53	2.42	1.38	0.46	1.27	0.68
*Tricuspid valve*							
*E*/*e*′	–	3.09	24.55	10.02	5.58	8.89	6.57

### Correlation between inferior vena cava variables and invasively measured central venous pressure (CVP_i_) (Table 4)

**Table 4:** Results of the linear regression between parameters of inferior vena cava and invasively measured central venous pressure

Variable	*R* (Pearson correlation coefficient)	*R* square	*P* value
Inferior vena cava systolic velocity	− 0.303	0.092	0.051
Inferior vena cava systolic velocity time integral	− 0.150	0.022	0.344
Inferior vena cava diastolic velocity	− 0.295	0.087	0.061
Inferior vena cava diastolic velocity time integral	− 0.203	0.041	0.203
Inferior vena cava reversal velocity	− 0.046	0.002	0.774
Inferior vena cava reversal velocity time integral	− 0.074	0.005	0.643
Inferior vena cava systolic to diastolic velocity ratio			
Inferior vena cava minimum size	0.336	0.113	0.0702
Inferior vena cava maximum size	0.333	0.111	0.031
			Central venous pressure = 9.04 + 0.23 × IVC max
Body surface area-indexed inferior vena cava minimum size	− 0.038	0.001	0.812
Body surface area-indexed inferior vena cava maximum size	− 0.083	0.007	0.602
Inferior vena cava collapsibility index	− 0.061	0.004	0.702
Inferior vena cava distensibility index	− 0.081	0.006	0.612
Inferior vena cava minimum size to descending aorta minimum size ratio $\left( {\frac{{{\text{IVC}}_{\min } }}{{{\text{DAO}}_{\min } }}} \right)$	0.448	0.201	0.004
			Central venous pressure = 8.51 + 2.39 × $\frac{{{\text{IVC}}_{\min } }}{{{\text{DAO}}_{\min } }}$
Inferior vena cava maximum size to descending aorta maximum size minimal dimension ratio $\left( {\frac{{{\text{IVC}}_{\max } }}{{{\text{DAO}}_{\max } }}} \right)$	0.529	0.280	< 0.001
			Central venous pressure = 7.92 + 2.82 × $\frac{{{\text{IVC}}_{\max } }}{{{\text{DAO}}_{\max } }}$

There was a significant and moderate correlation between the ratio of the maximum size of the IVC to the maximum size of the descending aorta at the same level in the subcostal view and CVP_i_ (*r* = 0.529, *P* value < 0.001). Similarly, a significant correlation was observed between the minimal diameters of these two vessels and invasively measured central venous pressure (*r* = 0.448, *P* value = 0.004). Additionally, a weak correlation was found between the maximum size of the IVC, measured during inspiration, and the CVP_i_ (*r* = 0.333, *P* value = 0.031). The corresponding regression equations are shown in Table 4.

### Correlation between superior vena cava variables and invasively measured central venous pressure (CVP_i_) (Table 5)

**Table 5:** Results of the linear regression between parameters of superior vena cava and invasively measured central venous pressure

Variable	*R* (Pearson correlation coefficient)	*R* square	*P* value
Superior vena cava systolic velocity	0.299	0.089	0.081
Superior vena cava systolic velocity time integral	− 0.207	0.043	0.232
Superior vena cava diastolic velocity	− 0.219	0.048	0.213
Superior vena cava diastolic velocity time integral	− 0.158	0.025	0.371
Superior vena cava reversal velocity	0.104	0.011	0.572
Superior vena cava reversal velocity time integral	0.044	0.002	0.810
Superior vena cava *S* velocity/*D* velocity ratio (SVC_S/D_)	0.462	0.214	0.006
			Estimated central venous pressure = 7.46 + 2.08 × SVC_S/D_

Pearson Chi-square or Fisher’s exact test	
Categorical variable	*P* value
SVC S/D < 1.9	0.394

A significant and moderate correlation was observed between the ratio of *S* velocity to *D* velocity (*r* = 0.462, *P* value = 0.006). Table 5 displays the regression equation corresponding to the aforementioned relationship.

### Correlation between hepatic vein variables and invasively measured central venous pressure (CVP_i_) (Table 6)

**Table 6:** Correlation between parameters of Doppler of hepatic vein and invasively measured central venous pressure

Linear regression			
Continuous Variables	*R* (Pearson correlation coefficient)	*R* square	*P* value
Hepatic vein retrograde flow velocity (A wave)	− 0.116	0.014	0.480
Hepatic vein retrograde flow velocity time integral (A wave)	− 0.080	0.006	0.630
Hepatic vein antegrade systolic velocity (S wave)	− 0.136	0.019	0.389
Hepatic vein antegrade systolic velocity time integral (S wave)	− 0.191	0.037	0.225
Hepatic vein antegrade diastolic velocity (D wave)	− 0.155	0.024	0.326
Hepatic vein antegrade diastolic velocity time integral (D wave)	− 0.143	0.020	0.366
Hepatic vein V Wave velocity	0.009	0.000	0.956
Hepatic vein V Wave velocity time integral	0.087	0.008	0.598
Hepatic vein maximal forward velocity	0.169	0.028	0.285
Hepatic vein minimal forward velocity	0.236	0.056	0.133
Hepatic vein systolic filling fraction	0.006	0.000	0.972
Hepatic vein systolic velocity to diastolic velocity ratio	0.052	0.003	0.748

Pearson Chi-square or Fisher’s exact test	
Categorical variables	*P* value
Hepatic vein systolic filling fraction > 55%	0.091
Hepatic vein systolic velocity/diastolic velocity ≤ 1	0.689

We did not observe any significant relationship between the Doppler variables of the hepatic vein and CVP_i_. Similarly, there was no significant correlation between a hepatic vein systolic filling fraction greater than 55% or a hepatic vein systolic velocity to diastolic velocity ratio of less than or equal to 1 and CVP_i_ (*P* values of 0.091 and 0.689, respectively).

### Correlation between tricuspid valve variables and invasively measured central venous pressure (CVP_i_) (Table 7)

**Table 7:** Correlation between tricuspid valve Doppler and tissue Doppler parameters and invasively measured central venous pressure

Linear regression			
Continuous variables	*R* (Pearson correlation coefficient)	*R* square	*P* value
Tricuspid valve E wave velocity	0.047	0.002	0.766
Tricuspid valve E wave velocity time integral	0.058	0.003	0.720
Tricuspid valve E wave acceleration time	0.007	0.000	0.965
Tricuspid valve E wave acceleration rate	0.080	0.006	0.621
Tricuspid valve E wave deceleration time	− 0.023	0.001	0.884
Tricuspid valve E wave deceleration rate	0.029	0.001	0.856
Tricuspid valve E wave duration	− 0.041	0.002	0.797
Tricuspid valve A wave velocity	0.226	0.051	0.150
Tricuspid valve A wave velocity time integral	0.093	0.009	0.562
Tricuspid valve A wave acceleration time	− 0.097	0.009	0.545
Tricuspid valve A wave acceleration rate (TV_AAR_)	0.315	0.099	0.048
			Regression equation = 9.9 + 0.001 × TV_AAR_
Tricuspid valve A wave deceleration time	− 0.156	0.024	0.324
Tricuspid valve A wave deceleration rate	0.264	0.07	0.09
Tricuspid valve A wave duration	− 0.221	0.159	0.159
Tricuspid valve e′ wave velocity	0.107	0.011	0.507
Tricuspid valve a′ wave velocity	− 0.055	0.005	0.734
Tricuspid valve isovolumic relaxation time	0.105	0.011	0.503
*E*/*e*′	0.018	0.000	0.913

Among the Doppler and tissue Doppler variables of the tricuspid valve, which included *E*/*e*′, only the tricuspid valve A wave acceleration rate (TVAAR) exhibited a weak correlation with CVP_i_ (*r* = 0.315, *P* value = 0.048). The corresponding regression equation can be found in Table 7.

### Correlation between right atrial variables and invasively measured central venous pressure (CVP_i_) (Table 8)

**Table 8:** Results of the linear regression between parameters of the right atrium and invasively measured central venous pressure

Variable	*R* (Pearson correlation coefficient)	*R* square	*P* value
Right atrial vertical (major) diameter (RA_VD_)	0.409	0.168	0.010
			Central venous pressure = 7.14 + 0.15 × RA_VD_
Right atrial horizontal (minor) diameter	0.232	0.054	0.155
Right atrial area (RA_A_)	0.384	0.148	0.014
			Central venous pressure = 8.73 + 0.41 × RA_A_
Right atrial volume	0.261	0.068	0.156
Body surface area-indexed right atrial volume	0.040	0.002	0.831

Fisher’s exact test	
Categorical variable	*P* value
Body surface area-indexed right atrial volume > 15 cm^3^/m^2^	1

A moderate correlation was observed between the major axis of the right atrial vertical axis and CVP_i_ (*r* = 0.409, *P* value = 0.010). Similarly, a weak relationship was found between the right atrial area and CVP_i_ (*r* = 0.384, *P* value = 0.014). The corresponding regression equations are displayed in Table 8.

### Introduction of a multivariable equation for estimation of central venous pressure (Fig. 3)

**Fig. 3:**
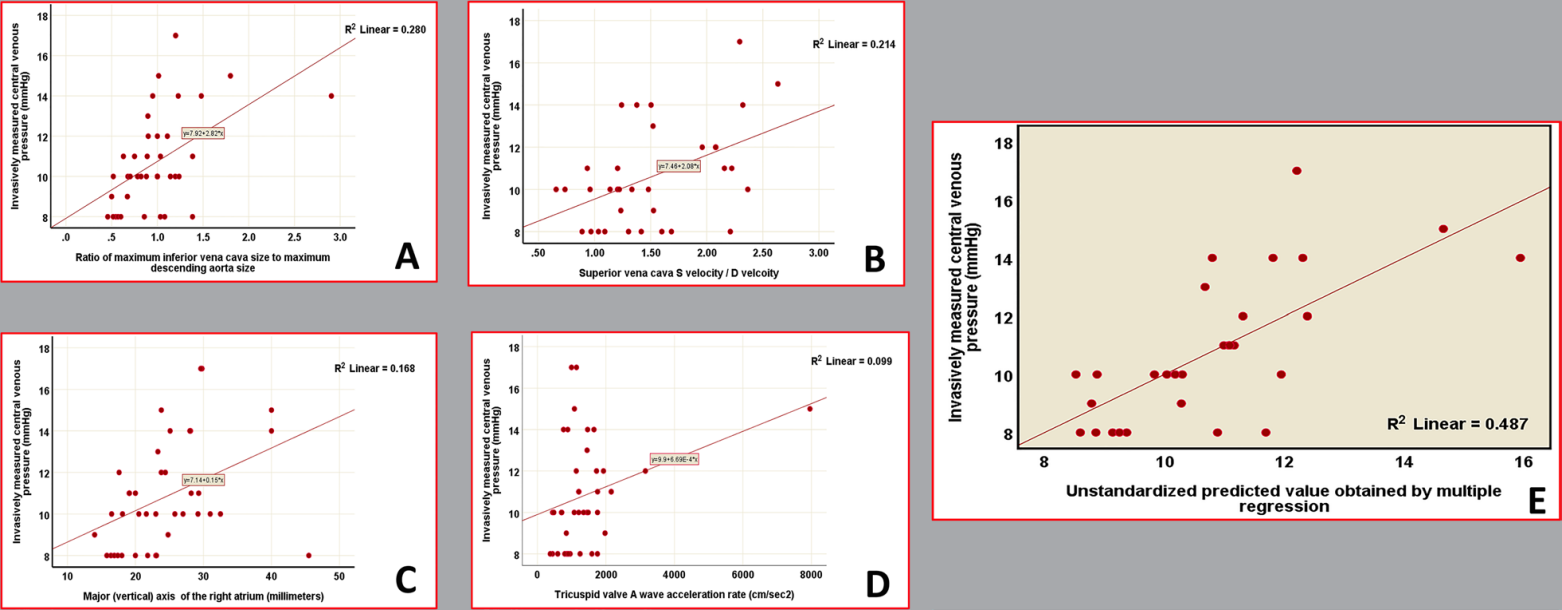
A scatterplot depicting the correlation between invasively measured central venous pressure and the ratio of the maximal diameter of the inferior vena cava (IVC) to the maximal diameter of the descending aorta. B Scatterplot illustrating the correlation between invasively measured central venous pressure and the superior vena cava S/D velocity ratio. C Scatterplot showing the correlation between invasively measured central venous pressure and the right atrial vertical axis. D Scatterplot demonstrating the correlation between invasively measured central venous pressure and the tricuspid valve A wave acceleration rate. E Scatterplot illustrating the correlation between invasively measured central venous pressure and multiple variables, including the ratio of the maximal diameter of the inferior vena cava (IVC) to the maximal diameter of the descending aorta, superior vena cava S/D velocity ratio, right atrial vertical axis, and tricuspid valve A wave acceleration rate

Using the most robust parameter related to IVC, SVC, tricuspid valve, and right atrium, we performed a multiple regression analysis and obtained the following multi-parametric equation.

With a strong correlation with CVP_i_ (Pearson *r* = 0.698, *P* value = 0.002):

$$\begin{aligned} {\text{Estimated CVP }}\left( {{\text{CVP}}e} \right) \, & = 4.36 + \left( {2.35 \times \frac{{{\text{IVC}}_{\max } }}{{{\text{DAO}}_{\max } }}} \right) \\ & \quad + \left( {1.06 \times {\text{SVC}}_{{\text{S/D}}} } \right) + \left( {0.059 \times {\text{RA}}_{{{\text{VD}}}} } \right) + \, \left( {0.001 \times {\text{TV}}_{{{\text{AAR}}}} } \right) \\ \end{aligned}$$

## Discussion

The right atrial cavity is anatomically connected to three vessels: the inferior vena cava (IVC), superior vena cava (SVC), and coronary sinus (CS). In a partially analogous manner to communicating vessels, the transmission of pressure from the right atrium to the IVC, SVC, and CS is anticipated to be influenced by several factors. These factors encompass the relative positioning of these vessels in relation to the right atrial cavity, the distance from the mid-right atrium, the discrepancy in the angle between the flow direction within these vessels and the right atrial cavity, and the size of the openings that establish the connection between these vessels and the right atrium (Fig. 1).

Among these vessels, the hepatic vein stands out as the furthest from the mid-right atrium and demonstrates a significantly distinct flow direction compared to the direction of flow within the right atrium. In our analysis, we did not identify any significant correlation between invasively measured central venous pressure and the hepatic vein systolic to diastolic velocity ratio or hepatic vein systolic filling fraction, as presented in Table 6. In contrast, Nagueh et al. conducted a study involving 35 adult patients with a wide range of diagnoses, including Wolff–Parkinson–White, mediastinal tumor, dilated cardiomyopathy, and other diseases. They demonstrated a correlation coefficient of 0.86 between hepatic vein systolic filling fraction (HVSFF) and mean right atrial pressure or central venous pressure [15]. The mean RA pressure for their patients varied from 2 to 28 mmHg. Discrepancies in the findings may be attributed to disparities in patient age, patient composition, right atrial pressures, and Doppler profile of hepatic veins in infants and children.

In contrast to the hepatic vein, we found a significant and moderate correlation between invasively measured central venous pressure and the ratio of the maximal and minimal dimensions of the inferior vena cava (IVC) to the maximal and minimal dimensions of the descending aorta, respectively. The utilization of ratios proves to be more reliable in the pediatric population. Additionally, within the initial 24 h following cardiothoracic surgery, Krastins and colleagues reported elevated intra-abdominal pressure in 66.67% of pediatric patients [16]. Kaptein and colleagues noted that the dimensions of the IVC and IVC collapsibility could be influenced by various factors, including increased intra-abdominal pressure and tricuspid regurgitation [17]. Therefore, the unique postoperative environment presents additional challenges when utilizing IVC size as a surrogate for right atrial pressure, primarily due to the impact of elevated intrabdominal pressure on the thin-walled IVC [16–18].

However, our analysis did not reveal any statistically significant correlation between the collapsibility index of the inferior vena cava (IVC) and the invasively measured central venous pressure (CVP_i_). This lack of correlation may be attributed to limited respiratory excursion in pediatric patients following cardiac surgery, which can be influenced by factors such as pain, diaphragmatic paresis, or other confounding variables affecting IVC collapsibility [14]. Similarly, in a study conducted by Kishiki et al., which included 60 children with congenital heart disease, no significant correlation was found between the inferior vena cava collapsibility index (IVCCI) and mean right atrial pressure [18]. However, they did observe a significant and moderate correlation between IVCCI and mean right atrial pressure when measured using three-dimensional echocardiography.

We also observed a moderate correlation between the *S*/*D* velocity ratio of the superior vena cava and CVP_i_. The superior vena cava acts as a conduit vessel situated higher than the right atrium and, given the effects of gravity, might be expected to be less responsive to pressure variations within the right atrial cavity compared to the inferior vena cava, which is located lower than the right atrium. In contrast to the study of o Murayama et al., we did not find any significant relationship between *S*/*D* < 1.9 and elevated central venous pressure (or mean right atrial pressure) [19]. There is a question regarding the role of the relative positions of the superior vena cava (SVC) and inferior vena cava (IVC) in relation to the right atrial cavity. The SVC, positioned as a conduit vessel above the right atrial cavity, raises uncertainty about its responsiveness to changes in right atrial pressure compared to the inferior vena cava, which benefits from gravity due to its lower position relative to the right atrial cavity. However, no studies have been conducted to address this question thus far. Furthermore, in the postoperative environment of the cardiac intensive care unit (CICU), greater variations in preload and afterload of the right heart are anticipated, potentially impacting the Doppler pattern of the superior vena cava.

This study showed that the right atrial vertical axis (major axis) and the right atrial area had a moderate correlation with CVP_i_. Patel et al. demonstrated a correlation between the three-dimensional right atrial volume index and the mean right atrial pressure [20]. The lack of relationship of the horizontal axis may implicate the importance of choosing a dimension that is in alignment with the direction of flow within the right atrium may be a more sensitive variable to serve as a surrogate for mean right atrial pressure.

## Limitations

The main limitations of this study are its relatively small sample size and the lack of invasively measured mean right atrial pressure. However, it is important to note that a majority of similar previous and concurrent studies have also enrolled a similar or even smaller number of patients [21–23]. With regard to the latter limitation, it can be assumed that mean right atrial pressure is equal to central venous pressure since there was no obstruction observed between the superior vena cava and the right atrium.

Additionally, our measurements were taken at a time when the patients exhibited stable hemodynamic states. We refrained from repeating these measurements under varying loading conditions, such as hypovolemia or volume overload, for two primary reasons: to eliminate potential confounding factors and to prevent imposing extra stress on postoperative children who were not in a state of hemodynamic stability. Therefore, to evaluate the impact of different loading conditions on the results, further study is recommended.

## Conclusions

This study introduced a novel multi-parametric regression equation that demonstrates a strong correlation with invasively measured central venous pressure in 43 infants and children with congenital heart disease during the initial 48 h following cardiac surgery in the cardiac intensive care unit. The equation incorporates the maximal dimension of the inferior vena cava relative to the maximal dimension of the descending aorta at the same level, the right atrial vertical axis, the S/D ratio of the pulse Doppler of superior vena cava, and the acceleration rate of the A wave of the tricuspid valve (estimated CVP = 4.36 + (2.35 × IVC_max_/DAO_max_) + (1.06 × SVC_S/D_) + (0.059 × RA_VD_) + (0.001 × TV_AAR_).

Conducting future studies applying this formula to a prospective population of pediatric patients with congenital heart disease is crucial in order to determine the mean difference between the predicted and actual pressures.

## Data Availability

The datasets used in the current study are available from the corresponding author upon reasonable request.

## Abbreviations

ASD: Atrial septal defect
BT: Blalock–Taussig
CS.: Coronary sinus
CVP: Central venous pressure
CVP_e_: Estimated (predicted) central venous pressure
CVP_i_: Invasively measured central venous pressure
HV: Hepatic vein
HVSFF: Hepatic vein systolic filling fraction
IQR: Interquartile range
IVC: Inferior vena cava
IVCCI: Inferior vena cava collapsibility index
IVCDI: Inferior vena cava distensibility index
IVC_max_: Maximal dimension of inferior vena cava
IVC_min_: Minimal dimension of inferior vena cava
LPA: Left pulmonary artery
PDA: Patent ductus arteriosus
RA: Right atrium
RPA: Right pulmonary artery
RV: Right ventricle
SD: Standard deviation
SPSS: Statistical Package for Social Sciences
SVC: Superior vena cava
SVCS/D: Ratio of S wave to D wave of superior vena cava
TVAAR: Acceleration rate of A wave of the tricuspid valve
